# Sequentially optimized reconstruction strategy: A meta-strategy for perimetry testing

**DOI:** 10.1371/journal.pone.0185049

**Published:** 2017-10-13

**Authors:** Şerife Seda Kucur, Raphael Sznitman

**Affiliations:** ARTORG Center for Biomedical Engineering Research, University of Bern, Bern, Switzerland; The University of Melbourne, AUSTRALIA

## Abstract

Perimetry testing is an automated method to measure visual function and is heavily used for diagnosing ophthalmic and neurological conditions. Its working principle is to sequentially query a subject about perceived light using different brightness levels at different *visual field* locations. At a given location, this query-patient-feedback process is expected to converge at a perceived sensitivity, such that a shown stimulus intensity is observed and reported 50% of the time. Given this inherently time-intensive and noisy process, fast testing strategies are necessary in order to measure existing regions more effectively and reliably. In this work, we present a novel *meta*-strategy which relies on the correlative nature of visual field locations in order to strongly reduce the necessary number of locations that need to be examined. To do this, we sequentially determine locations that most effectively reduce visual field estimation errors in an initial training phase. We then exploit these locations at examination time and show that our approach can easily be combined with existing perceived sensitivity estimation schemes to speed up the examinations. Compared to state-of-the-art strategies, our approach shows marked performance gains with a better accuracy-speed trade-off regime for both mixed and sub-populations.

## 1 Introduction

**Fig 1:**
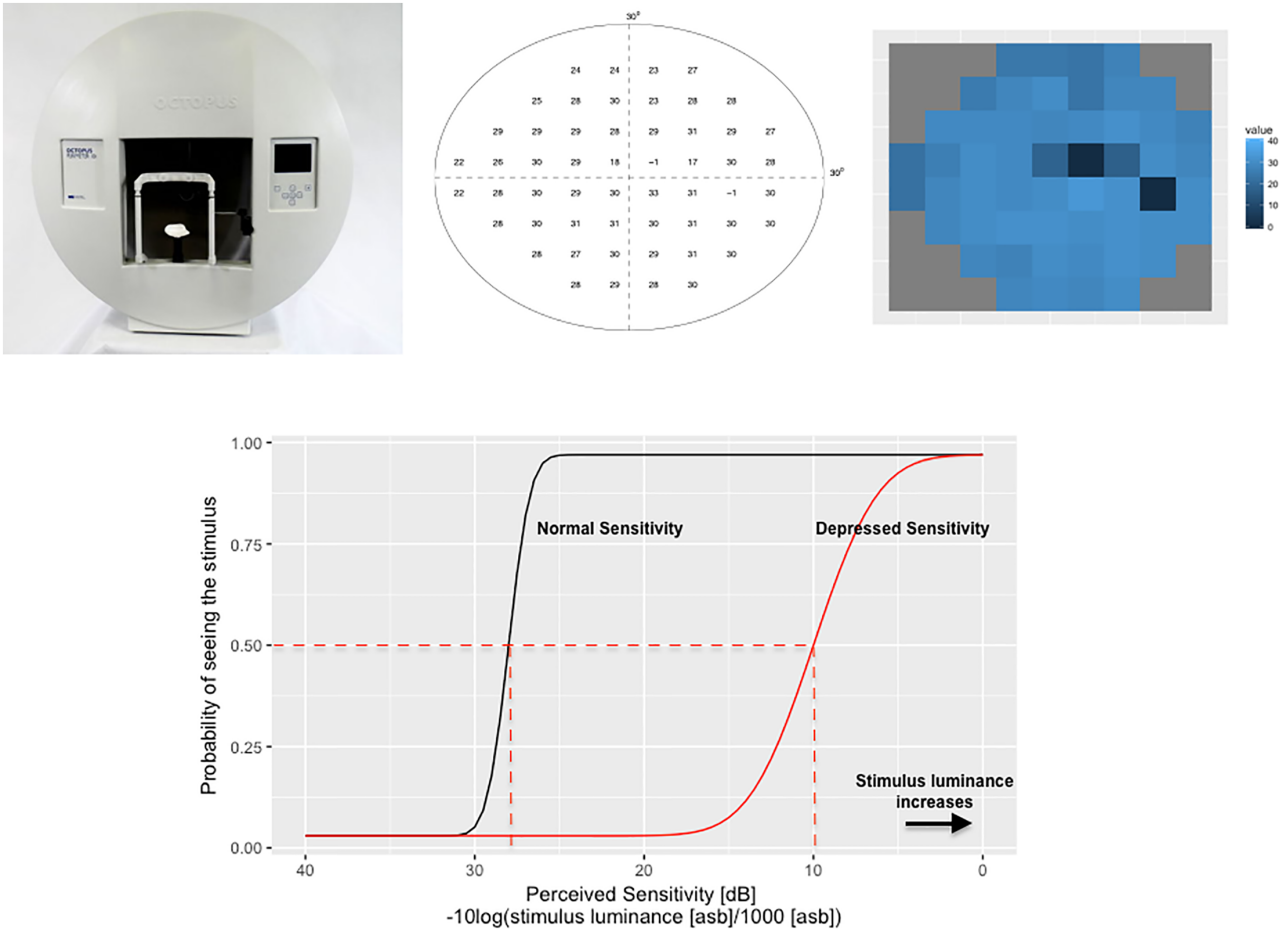
Perimetry testing and visual field. (left) A perimetry device, (center) a visual field with perceived sensitivities (PS) at locations in the central 30° field and (right) the associated image representation. Dark regions correspond to visual defects. (bottom) Probability-of-seeing-curve. The probability of seeing a stimulus increases with increasing stimulus luminance. Note the inverse relationship between sensitivity and stimulus luminance.

Standard Automated Perimetry (SAP) is one of the most commonly used techniques for measuring a subject’s perceived visual ability. For a given eye, it provides quantitative measurements of visual function represented as a two-dimensional spatial visual field map (see Fig 1). As a medical imaging system, it is of great clinical importance for diagnosing and monitoring numerous ophthalmic diseases (e.g., glaucoma) and for detecting neurological conditions [1, 2].

At its core, the goal of SAP is to determine at each location of the visual field the perceived sensitivity (PS), i.e., the brightness level with which a subject sees a stimulus 50% of the time. Using a perimeter as the one shown in Fig 1 (left), this is achieved using a semi-automated query-response procedure: while fixating their gaze at a central point on a screen, a subject is presented with light stimuli of adaptively selected brightness at different locations of the visual field and is asked to press a button whenever the stimulus is perceived. As such, the responses of subjects are inherently noisy and response reliability reduces over time due to fatigue effect [3, 4]. While presenting all brightness levels at all locations multiple times would provide many responses and allow one to average out response noise, doing so would be extremely time consuming (i.e., more than 15 minutes per eye [3]), further worsening the induced fatigue-bias. Conversely, testing one stimulus at a handful of locations would produce highly inaccurate visual fields and be ill-suited for clinical use. As such, a central goal of SAP testing strategies is to optimize in which order and how often the locations should be tested in order to be both fast and accurate [5].

A number of SAP strategies have been introduced in the literature and are now common in manufactured devices. They commonly rely on staircasing schemes [6] as in the Dynamic Test Strategy (DTS) [7] and in Tendency Oriented Perimetry (TOP) [8] where the intensity of presented stimuli changes by fixed or adaptive step sizes according to the patient responses. Alternative methods have also been introduced such as the Zippy Estimation by Sequential Testing (ZEST) [6, 9], where the next stimulus is determined by leveraging patient responses within a Bayesian model. Simiarly, the widely popular SITA algorithm [5, 10] and its faster counterpart SITA FAST [11], also follow Bayesian schemes that periodically update probability distributions as new locations are tested. While the above methods are commonly used in clinics, their performance could be improved.

More recently, [12, 13] focused on spatial models where neighboring information is exploited in a customized or data-driven manner. These approaches have been shown to lead to similar or better accuracy than ZEST. However, they typically keep the test time either the same [13] or only bring speed improvement in healthy subjects [12]. A more recent attempt to improve speed-accuracy trade-off has been presented in [14] where a graphical model of the visual field was presented and allows response information to propagate during an examination leading to shorter test time. This strategy however is sensitive to the selection of model parameters and therefore relies on a meticulous optimization procedure, making it ill-suited for clinical use. A parameter-free, easy-to-implement, fast and accurate strategy is preferable from both a clinical and manufacturer point of view.

Towards this end, we introduce a novel *meta*-strategy that leads to important reductions in examination time, by speeding up existing perimetry strategies. Our new meta-strategy, namely Sequentially Optimized Reconstruction Strategy (SORS), is inspired by compressed sensing [15] and sparse approximation [16] methods for signal and image reconstruction. Since previous studies have shown that testing optimal subsets of locations can lead to accurate assessment of glaucomatous defects [17, 18], we propose to *reconstruct* visual fields from a limited number of measurements i.e., testing a sparser grid of test locations, by assuming the existence of correlations between visual field locations. During an initial training phase, our method sequentially estimates the order in which different locations should be tested to reconstruct visual fields most accurately. At examination time, locations are sequentially tested in the found order using a standard strategy, from which the visual field is reconstructed after each tested location. Reconstructed locations are then used as starting estimates when querying following test locations. In particular, we claim that our meta-strategy can be used and be beneficial using a ZEST Bayesian scheme or DTS staircasing. We show experimentally on a visual field data set of both healthy and glaucomatous subjects, that our strategy provides large speed gains compared to existing methods without compromising the accuracy of estimated visual fields. In addition, we show that although our strategy does not require all locations to be tested, it allows for good accuracy even in cases of local visual impairment.

The remainder of this article is organized as follows: In Sec. 1, we summarize existing perimetry testing strategies. We then outline the training and testing phases of the SORS method in Sec. 3. Experimental validation of our method is then outlined in Sec. 4 and concluding remarks are given in Sec. 5.

## 2 Related work

In this section, we first summarize fundamentals in perimetry and describe a number of related perimetry testing strategies.

The goal of perimetry is to estimate the PS at *M* locations (e.g., *M* = 54 as in Fig 1 (middle)) describing the visual field. The PS at an individual location corresponds to the sensitivity, in dB, for which there is a 50% probability chance of being observed. Traditionally, this has been modeled using a *probability-of-seeing-curve* (POSC) [3, 19] such as the one illustrated in Fig 1 (bottom). As such, the distribution of responses is of maximum entropy, as the likelihood of observing an incorrect response (i.e., a false positive or false negative) is maximal at the PS value. In addition, at unhealthy locations with lower PS, the number of incorrect answers is expected to increase as the POSC becomes more gradual (e.g., red curve in Fig 1 (bottom)).

To estimate visual fields using SAP, different automated methods have been proposed in the past. Each of them include the following: (1) a method to determine what initial intensity should be shown when testing a given location, (2) a local PS testing strategy that determines what intensities should be presented over time at a given location and (3) a strategy for selecting the order in which different locations are evaluated.

From this, a number of methods have been proposed in order to produce accurate or approximate visual fields. The simplest method is the Full Threshold (FT) strategy [5]. It evaluates each location using a predefined staircase intensity sequence (e.g., increase or decrease the intensity based on the previous response). After initially testing four anchor points starting from population normal values, it tests subsequent locations by updating the initial stimulus based on previously tested neighbors [3]. FT leads to accurate visual field estimates for normal subjects as it presents many stimuli but inevitably leads to higher examination times, ranging from 12 to 18 minutes per eye [3]. A variation of FT is DTS which uses staircasing with adaptive step sizes that are determined by the slope of the POSC. Accordingly, larger step sizes are used for depressed PS values where the POSC is shallower. All locations are tested but each starting intensity is based on a local average of found PS values. In general, DTS reduces testing time on average by 40% compared to FT with a reasonable visual field approximation [3] and is a standard of care in many eye clinics and hospitals. TOP [3] on the other hand uses an asynchronious staircasing approach with deterministic steps at individual locations such that each location is only tested once. Locations in groups of four are tested group by group; once one group of test locations is evaluated, the estimates of the locations in the other groups are updated by averaging the estimates at their already-tested-neighboring locations. The updated estimates are then used as the starting points for querying the next group of locations. As TOP only presents one stimulus per location, it results in a very fast but error-prone estimation procedure.

An alternative is ZEST [9], which unlike FT, avoids a predefined staircase and opts for a sequential Bayesian model to select likely PS values. As such, it highly depends on a probability mass function (PMF) over the PS values for a given location in order to compute posterior distributions of PS. ZEST evaluates all visual field locations in a random order, yet has been found to effectively reduce the number of presentations thanks to the Bayesian principle [20]. Also using this Bayesian principle, SITA [5, 10] and SITA FAST [11] are broadly used methods and have been reported to perform comparably with DTS and TOP, respectively. While technical details concerning either SITA and SITA FAST remain unavailable, both methods display advantages and drawbacks over TOP and DTS [21–24].

One common aspect of the presented approaches so far is that they test every location at least once. In contrast, Spatial Entropy Pursuit (SEP) [14] combines the ZEST method and a graphical model to reduce the examination time. It uses a combined entropy and gradient heuristic to adaptively select which locations should be tested within a probabilistic model. In addition, unlike previously mentioned strategies, it is able to ignore some locations that are deemed certain even though they have not been explicitly tested. SEP is reported to reduce the number of stimuli by 55% for healthy subjects and by 23% for glaucomatous subjects when compared to DTS. A limitation of SEP however is its sensitivity to the selected graphical model and ZEST parameters. It therefore requires a rigorous parameter optimization to perform at an effective level.

Overall, while some of the aforementioned methods are used in clinical care (i.e., SITA, DTS, FT and TOP), they could be improved in terms of speed and accuracy. To overcome this, we propose a meta-strategy, capable of using traditional staircase methods or ZEST-like Bayesian strategies at individual locations but in a more efficient and faster manner. Our approach, in essence, determines which locations should be chosen and in what order they should be evaluated in order to maximally improve the visual field estimate in the least amount of time. As we show in our experiments, SORS brings a large improvement when compared to existing methods in terms of speed, while suffering less from estimate errors.

## 3 Sequentially optimized reconstruction strategy

We now describe our method, SORS, which treats the problem of visual field estimation as a reconstruction problem from sparse observations. In this setting, the observations will be a small or limited number of visual field locations that have been viewed to a satisfactory accuracy using either a traditional staircasing or a Bayesian method. Using these locations and their values, we will leverage the correlative nature of the locations within a training data set to estimate the PS at unobserved locations of the visual field. As such, SORS can be split into two sections:

1. Training phase: From a data set of fully observed visual fields, we will determine which locations are most effective to reconstruct the entire visual field from partial observations and simultaneously compute optimal reconstruction coefficients. This will be performed for an increasing number of observed locations in a greedy manner.
2. Examination phase: For a new examination, found locations and reconstruction coefficients will be used to infer unobserved locations. If the user prefers a more accurate estimate, further locations can be observed using previously estimated PSs as starting points and the reconstruction can be recomputed.

We now specify some notation that will be necessary throughout the remainder of the paper.

### 3.1 Notation

Let $X\in R^{M\times N}$ be a matrix of *N* visual fields where the *n*th column vector, $x_{n}\in R^{M},n=1,..,N$, corresponds to a visual field with *M* PS values. The ordering of visual field locations is kept constant for all *N* samples and is denoted by the sequence Ω = [1, …, *M*]. While Ω is a sequence, we will slightly abuse this notation and use set operators on Ω as well. We define *S* ≤ *M* to be the number of observed visual field locations tolerated during an examination and let Ω_*S*_ ∈ Ω be the sequence of such observed location indices. Our assumption is that ∀*n*, *x*_*n*_ can be estimated by a linear combination of its observed entries using a basis matrix $D\in R^{M\times S}$ that defines the linear relationship between test locations.

### 3.2 Training phase

Assuming that PS values at different locations are linearly-dependent to each other and that an examination allows for up to *S* observations to be made, we can approximate the training set *X* by computing

$$\begin{array}{c} \hat{X}=DY_{\Omega_{S}}, \end{array}$$ (1)

where $\hat{X}$ is an approximate reconstruction of the visual fields *X* and *Y*_Ω_*S*__ = *I*_Ω_*S*__ *X* such that

$$\begin{array}{c} {\left(I_{\Omega_{S}}\right)}_{i,j}=\left\{ \begin{array}{cc} 1 & \text{if}{\left(\Omega_{S}\right)}_{i}={\left(\Omega \right)}_{j},\\ 0 & \text{otherwise,} \end{array}\right. \end{array}$$ (2)

where $I_{\Omega_{S}}\in {\text{I}\;\text{R}}^{S\times M}$ and (Ω_*S*_)_*i*_ = (Ω)_*j*_ indicates that the *i*th measurement corresponds to the location *j*. By this, the measurement matrix *Y*_Ω_*s*__ is a sub-matrix of *X* consisting of rows indexed by Ω_*S*_.

Recall that we are interested in finding an optimal sequence of *S* locations to evaluate and a corresponding basis that would lead to a good estimate $\hat{X}$. We thus cast this as an optimization problem of the following form,

$$\begin{array}{c} \left\{D^{*},\Omega_{S}^{*}\right\}=\underset{\begin{array}{c} D\in \text{I}\;{\text{R}}^{M\times S},\\ \Omega_{S}\in \Omega \end{array}}{\text{arg min}}{||X-DY_{\Omega_{S}}\left|\right|}_{2}^{2}. \end{array}$$ (3)

Note that solving Eq (3) by brute-force suggests optimizing iteratively over *D* for every possible sequence Ω_*S*_, which is not feasible as the number of available sequences could be very large depending on *S*.

**Fig 2:**
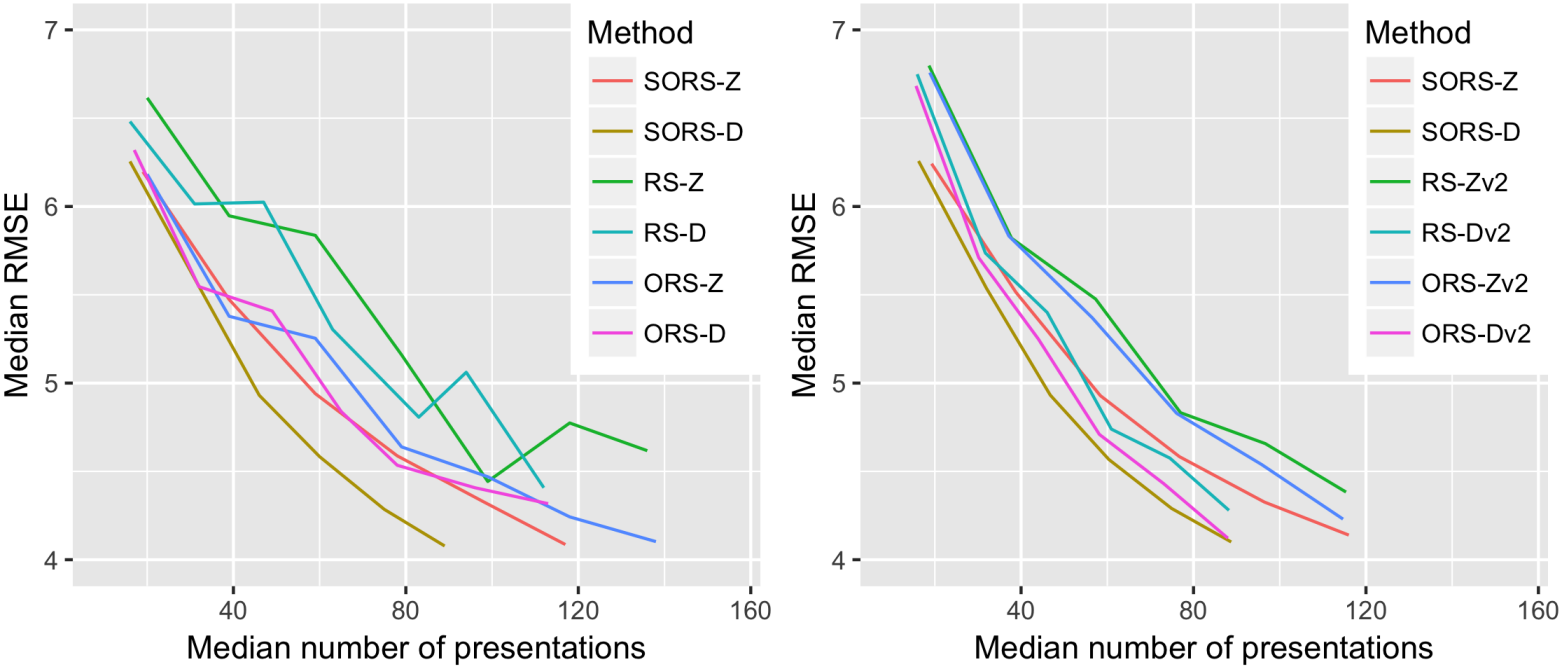
Performance comparison between SORS and alternative optimization schemes, namely Reconstruction Strategy (RS) and Optimized Reconstruction Strategy (ORS). We present one version of RS and ORS where there is no intermediate reconstruction step in test time (left) and on the a second version where intermediate reconstruction steps were incorporated, called RSv2 and ORSv2 (right). Figures show the median Root Mean Square Error (RMSE) performances of each method with respect to the median number of stimuli presentations. See Appendix A for details.

Alternatively, we propose a greedy approach which searches for a good subset Ω_*S*_ by sequentially selecting locations rather than trying to find them in one step. Formally, the *k*th element in $\Omega_{S}=\left\{l_{1}^{*},l_{2}^{*},...,l_{S}^{*}\right\}$ is found by

$$\begin{array}{c} l_{k}^{*}=\underset{l\in \Omega \setminus \Omega_{k-1}}{\text{arg min}}{||X-D_{k}^{l}Y_{\Omega_{k-1,l}}\left|\right|}_{2}^{2},\;k=1,\ldots ,S, \end{array}$$ (4)

where

$$\begin{array}{c} D_{k}^{l}=XY_{\Omega_{k-1,l}}^{T}{\left(Y_{\Omega_{k-1,l}}Y_{\Omega_{k-1,l}}^{T}\right)}^{-1}, \end{array}$$ (5)

is a basis matrix associated with the measurement matrix *Y*_Ω_*k*−1,*l*__, Ω_*k*−1,*l*_ is the sequence Ω_*k*−1_ to which location *l* is appended at the end and Ω_0_ = ∅. As the intermediate basis matrices will be also used at examination time, the procedure results in both the sequence $\Omega_{S}^{*}=\left\{l_{1}^{*},l_{2}^{*},...,l_{S}^{*}\right\}$ and the corresponding basis set $D^{*}=\left\{D_{k}^{l_{k}^{*}}|k=1,2,...,S\right\}$. We summarize the training phase algorithm of SORS in Alg. 1. While the presented greedy approach presumably leads to sub-optimal solution, we show in Appendix A that it provides superior performances over potential alternative schemes (see Fig 2).

**Algorithm 1:** SORS Training algorithm

 **Input**: Training data *X*, location set Ω, *S*

1 Initialize $\Omega_{S}^{*}=\emptyset ,D^{*}=\emptyset ,\Omega_{0}=\emptyset ,I_{\Omega_{0}}=0$;

2 **for** *k* = 1, 2, …, *S* **do**

3  *error*_*l*_ ← 0, ∀*l* ∈ (Ω\Ω_*S*_)

4  **for** *l* ∈ (Ω\Ω_*S*_) **do**

5   Ω_*k*−1,*l*_ ← Ω_*k*−1_∪{*l*}

6   *Y*_Ω_*k*−1,*l*__ ← *I*_Ω_*k*−1,*l*__ *X*

7   $D_{k}^{l}\leftarrow XY_{\Omega_{k-1,l}}^{T}{\left(Y_{\Omega_{k-1,l}}Y_{\Omega_{k-1,l}}^{T}\right)}^{-1}$

8   $\hat{X}\leftarrow D_{k}^{l}Y_{\Omega_{k-1,l}}$

9   $error_{l}\leftarrow {||X-\hat{X}\left|\right|}_{2}^{2}$

10  **end**

11  $l_{k}^{*}\leftarrow {\text{arg min}}_{l}error_{l}$

12  $\Omega_{S}^{*}\leftarrow \Omega_{S}^{*}\cup l_{k}^{*}$

13  $D^{*}\leftarrow D^{*}\cup D_{k}^{l_{k}^{*}}\;\text{with}\;D_{k}^{l_{k}^{*}}=XY_{\Omega_{S}^{*}}^{T}{\left(Y_{\Omega_{S}^{*}}Y_{\Omega_{S}^{*}}^{T}\right)}^{-1}$

14 **end**

 **Result:** Sequence $\Omega_{S}^{*}$, Basis set $D^{*}$

### 3.3 Examination phase

During an examination, the location ordering $\Omega_{S}^{*}$ is sequentially evaluated using either the staircasing or Bayesian approach for PS estimation. In the following, we detail this process and state how either location testing strategy can be used.

In general, we perform the following two steps iteratively for *S* locations using either PS estimation method, which we denote here as P:

1. Location *k* ∈ [1, *S*], $l_{k}^{*}$ of an unknown visual field *e* is tested with P and the entire visual field is reconstructed using the corresponding basis, $D_{k}^{l_{k}^{*}}$ as given by
  $$\begin{array}{c} {\hat{e}}_{k}=D_{k}^{l_{k}^{*}}y_{\Omega_{k}^{*}}, \end{array}$$ (6)
  where $y_{\Omega_{k}^{*}}$ is the observed measurement vector including all previous measurements at the locations $l_{1}^{*},l_{2}^{*},...,l_{k-1}^{*}$ as well as at the last one, i.e., $l_{k}^{*}$ and ${\hat{e}}_{k}$ is the estimated visual field at the *k*th step. Note that all the previously tested *k* PS values are used for this reconstruction step.
2. The starting intensity level for method P is updated at the unobserved location $l_{k+1}^{*}$ that is to be tested next using ${\hat{e}}_{k}$. As this process depends explicitly on P, we outline this more clearly for both staircasing and Bayesian methods below.

This two-step iterative process is stopped when all locations in $\Omega_{S}^{*}$ have been tested using P. Note that by updating the starting points for the next locations to query, we are able to further reduce the number of stimuli presentations at a given location, as the presented stimulus is on average closer to the true PS value. We now detail two versions of our method that use different PS estimation strategies.

#### 3.3.1 SORS-ZEST

This version of SORS uses the ZEST Bayesian procedure when testing a single test location. As previously mentioned, ZEST starts testing a location according to a prior PMF which is a weighted combination of normal and abnormal PS as described in [20]. In practice, this corresponds to a mixture of two Gaussian distributions centered on an age-matched normal value and on an abnormal value (0 in practice), representing healthy and glaucomatous population, respectively. This can be formulated as

$$\begin{array}{c} {\text{PMF}}^{l}\approx G\left(nv_{l},\sigma_{l}\right)+\alpha G\left(0,1\right)+\epsilon_{l}, \end{array}$$ (7)

where PMF^*l*^ is the PMF at location *l*, *G*(*μ*, *σ*) is a Gaussian function with mean *μ* and standard deviation deviation *σ*, *nv*_*l*_ is the age-matched normative value associated with location *l*, *α* is the weight of the Gaussian function corresponding to sick population, and *ϵ*_*l*_ is a bias term to guarantee that no value is assigned zero probability.

Given that in step 2 of the examination method, we can reconstruct visual fields from few observations using $D_{k}^{l_{k}^{*}}$, we propose an alternative prior distribution for the next location to be tested, created by shifting *G*(*nv*_*l*_, *σ*_*l*_) such that its mode is given by the estimated value at the location $l_{k+1}^{*}$. That is, we let

$$\begin{array}{c} {\text{PMF}}^{l_{k+1}^{*}}\approx G\left({\hat{e}}_{k}^{l_{k+1}^{*}},\sigma_{l}^{2}\right)+\alpha G\left(0,1\right)+\epsilon_{l}, \end{array}$$ (8)

where ${\text{PMF}}^{l_{k+1}^{*}}$ is the prior PMF associated with location $l_{k+1}^{*}$ and ${\hat{e}}_{k}^{l_{k+1}^{*}}$ is the estimated value at the $l_{k+1}^{*}$ location of the last reconstructed visual field ${\hat{e}}_{k}$. Note that the first test location has a standard prior PMF as given in Eq (7) but that the following locations have adjusted PMFs according to the reconstructed visual field.

#### 3.3.2 SORS-Dynamic

In this version of SORS, we use a staircasing approach with step sizes that adapt to the slope of POSC as in DTS. As we locally use the same procedure as DTS, we denote this version SORS-Dynamic where SORS mainly differs from DTS in the selection of locations to test, in the determination of the starting stimulus luminance and most importantly, in the number of test locations queried. In this method, the starting stimulus presented at the next location $l_{k+1}^{*}$ is given by ${\hat{e}}_{k}^{l_{k+1}^{*}}-\tau$ estimated during the *k*th reconstruction step. Note that we set *τ* = 4Â dB for all experiments performed, as including this small offset provides superior performances in practice.

## 4 Results

### 4.1 Experimental set-up

We validated our approach using a publicly available visual field data set [25, 26] containing 5108 visual fields from both eyes of 22 healthy and 139 glaucomatous patients. The data was collected using a Humphrey Visual Field Analyzer II (Carl Zeiss Meditec AG, Germany). Each visual field contains *M* = 54 test locations.

To evaluate the performance of SORS in comparison to established methods, we compare our method with ZEST [9], TOP [8], DTS [7] and SEP [14]. All experiments were implemented using R and the Open Perimetry Interface (OPI) [27, 28], which allows us to simulate the response of individuals according to their true visual field [12, 13, 29].

We performed a 10-fold cross-validation; training and test visual fields in each fold were selected such that they do not include visual fields from the same patient. That led to folds with roughly 4597 training and 511 test samples. For each fold, the optimal sequence of test locations $\Omega_{S}^{*}$, as well as the corresponding basis set $D^{*}$ were found for *S* = 1, 2, …, 40 and evaluated on the test data. In addition, for each fold, we optimized the ZEST parameters related to the prior probability of each location, specifically *σ*_*l*_ and *ϵ*_*l*_, while setting *α* to 0.1 in Eq (7). We set the ZEST stopping criterion as the standard deviation of the posterior PMF being less than 2 and the maximum number of stimuli per location being 4. False positive and false negative response rates of the simulated subjects were set to 0.03 and 0.01, respectively. Below, we present the results for one fold selected at random, as similar trends were observed in other folds.

### 4.2 Qualitative evaluation

**Fig 3:**
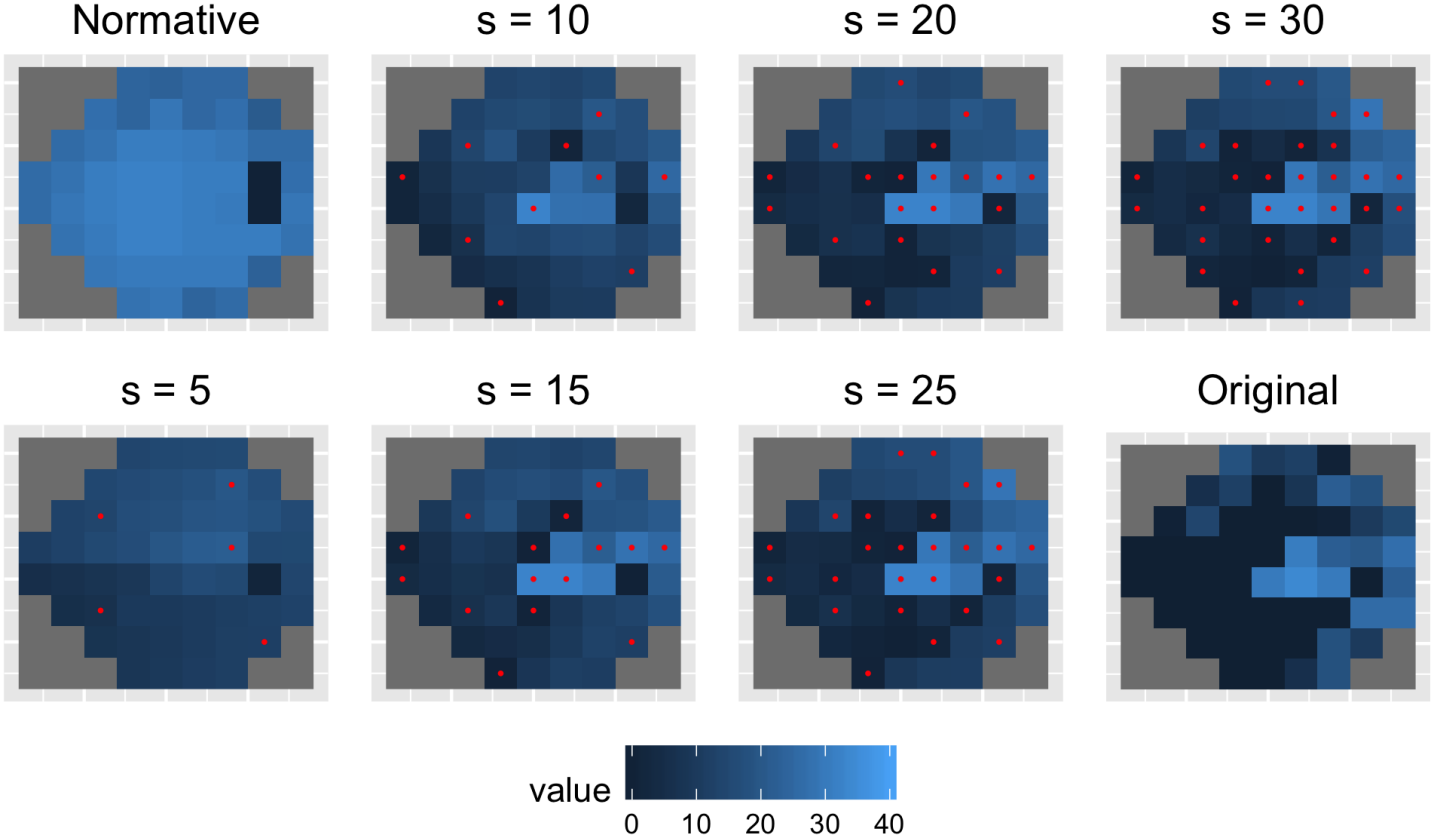
Qualitative evaluation of SORS. Top left shows the starting visual field with age-normalized values. Bottom right shows the true visual field to be estimated. In between, the sequentially estimated visual fields using *S* ∈ {5, 10, 15, 20, 25, 30} location measurements. Red points show the corresponding *S* tested locations.

We first show in Fig 3 an example of an examination and how SORS sequentially evaluates different locations. In each field, PS values are estimated (dark regions indicating defects) and red dots show tested locations. As more test locations are used, differences between the true and estimated PS values decrease and a reasonable estimation is achieved with only 15-20 locations tested. Note that even if not all locations are evaluated, the visual field estimate is close to the true visual field (see *S* = 25).

**Fig 4:**
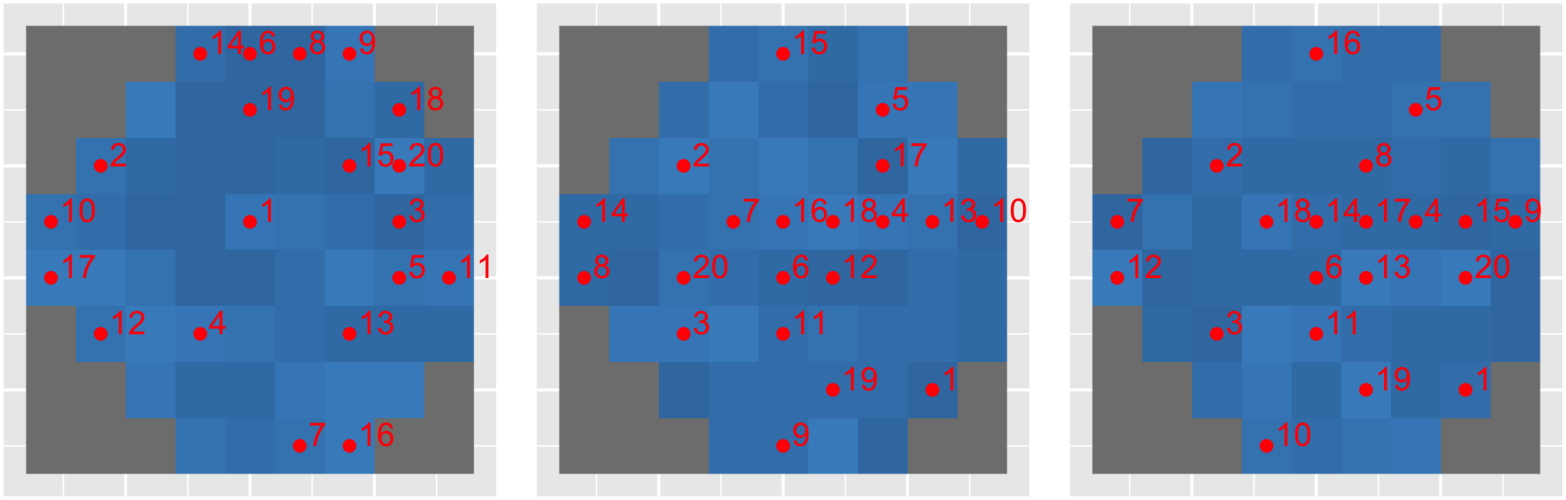
Optimal test locations found by SORS. Optimal test locations when trained on healthy (left), glaucomatous (middle) and mixed population (right) are presented. Numbers show the order in which the locations are evaluated.

Similarly, Fig 4 depicts the order of the 20 first locations selected as a function of the training set used. In particular, we show different orderings found when training using only healthy subjects (left), glaucoma patients (middle) and a mixed population of both subjects (right). Note that the mixed population ordering is similar to that of the glaucoma patient ordering, because the number of healthy subjects is an order of magnitude smaller than that of glaucoma patients in the mixed population. Importantly, there is a significant differences in selected locations between healthy and glaucomatous individuals. It can be seen that training on healthy subjects leads to more locations selected at the periphery of the visual field. This is in strong contrast to a concentrated set of central locations when training with glaucomatous subjects.

### 4.3 Accuracy and speed performance comparison

**Fig 5:**
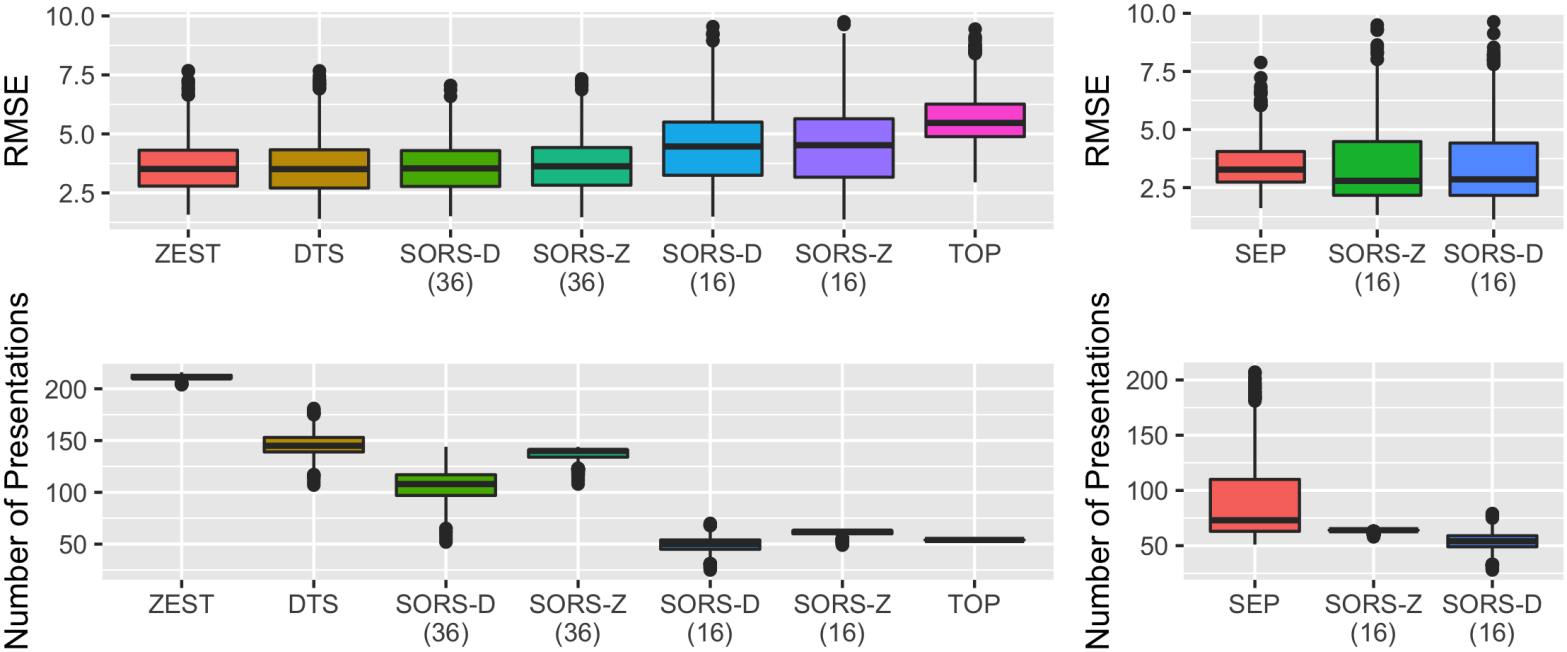
Performance benchmarking with the state-of-the-art perimetry strategies. SORS is compared to (left) existing and commercially used methods, (right) to SEP on mixed population. SORS is evaluated on 16 and 36 locations as specified in parenthesis. SORS-D and SORS-Z stand for SORS-Dynamic and SORS-ZEST, respectively.

Fig 5 presents quantitative performances of the evaluated methods in terms of Root Mean Square Error (RMSE) and the number of stimuli presentations used (i.e., examination time). Throughout the rest of the paper, we will use abbreviations SORS-D and SORS-Z which stand for SORS-Dynamic and SORS-ZEST, respectively.

Fig 5 (left) compares the performance of SORS with *S* = 16 and *S* = 36 with that of state-of-the-art strategies. With 54 stimuli presentations, TOP achieves relatively low accuracy (median RMSE of 5.47). Testing only 16 locations, SORS-D (median RMSE of 4.47, median number of presentations of 50) performs significantly better than TOP in both accuracy and speed (Mann-Whitney U test, *p* < 0.0001). Similarly, SORS-Z testing only 16 locations (median RMSE of 4.52, median number of presentations of 62) has a reduced RMSE compared to TOP (significant difference, Mann-Whitney U test, *p* < 0.0001), with a slightly higher number of presentations.

**Fig 6:**
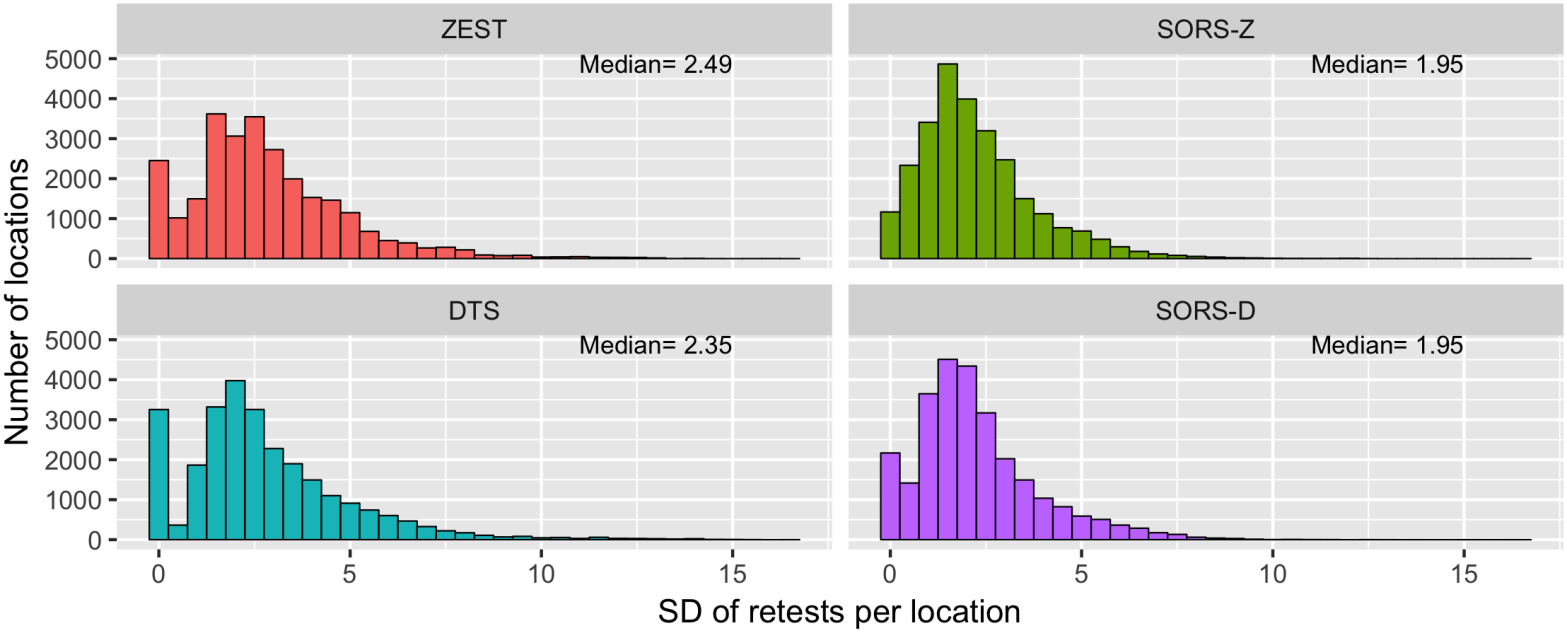
Test-retest variability of perimetry strategies. Standard deviations (SDs) of PST estimations of 5 tests per location are presented and the median of each distribution is shown in the top right corner. SORS approaches tested 36 locations.

**Fig 7:**
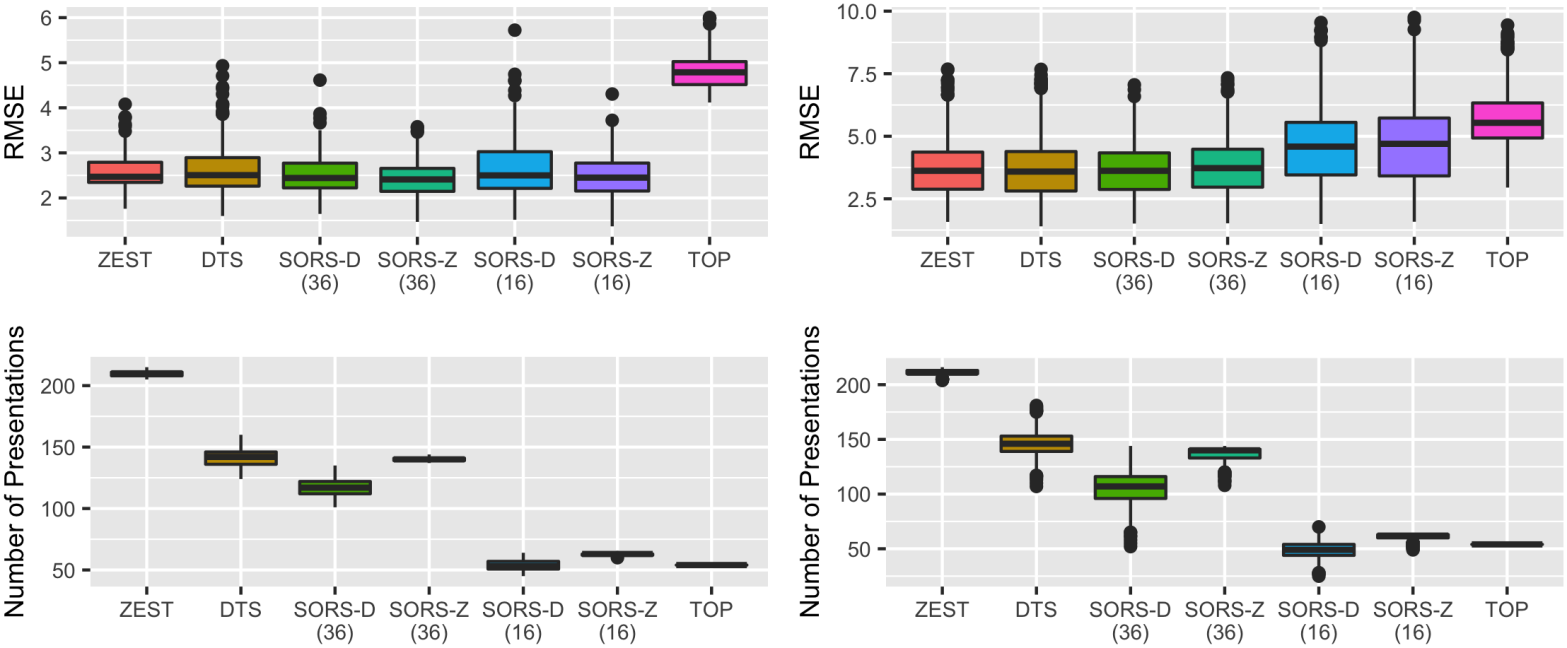
Performance comparison of perimetry strategies on different sub-populations. We present SORS performance on healthy (left) and glaucomatous (right) visual fields compared to state-of-the-art methods.

Testing 36 locations, SORS-D (median RMSE of 3.54) and SORS-Z (median RMSE of 3.63) achieve similar performance to DTS (median RMSE of 3.51, non-significant difference with SORS-D, Mann-Whitney U test, *p* > 0.05, significant difference with SORS-Z, Mann-Whitney U test, *p* < 0.001) and ZEST (median RMSE of 3.51, non-significant difference, Mann-Whitney U test, *p* > 0.05). At similar visual field estimate accuracy, SORS methods require fewer stimuli presentations than DTS and ZEST. More specifically, when compared to ZEST (median number of presentations of 211), SORS-Z (median number of presentations of 140) achieves the same accuracy (non-significant difference, Mann-Whitney U test, *p* > 0.05) with approximately 34% fewer number of stimuli presentations. Similarly, SORS-D (median number of stimuli presentations 108) achieves the same RMSE performance (non-significant difference, Mann-Whitney U test, *p* > 0.05) with DTS (median number of stimuli presentations 145) by reducing 25% of the required stimuli presentations (significant difference, Mann-Whitney U test, *p* < 0.0001). These results support the fact that SORS can speed up examinations more than other state-of-the-art approaches. In addition, our methods have less variance in the produced visual fields as evaluated in test-retest experiments (see Fig 6 and Appendix B) and perform well when testing on only healthy or glaucomatous populations (see Fig 7 and Appendix C).

To fairly compare SORS to SEP, we run experiments on the same training and test sets that were used in [14] and show the results in Fig 5 (right). First, one should note that as the test data set in this experimental set-up has 245 healthy and 172 glaucomatous visual fields, SORS-Z (median RMSE of 2.79 and median number of stimuli presentations of 64) and SORS-D (median RMSE of 2.85 and median number of stimuli presentations of 54) have lower RMSE and number of stimuli presentations than that shown in Fig 5 (left) where test set includes 32 healthy and 465 glaucomatous visual fields. Accordingly, when testing 16 locations, SORS-Z and SORS-D yield on average more accurate and faster examinations than SEP (median RMSE of 3.27 and median number of stimuli presentations of 73, significant difference, Mann-Whitney U test, *p* < 0.0001). In addition, the comparison between SEP and SORS-Z is interesting as they can both be seen as meta-strategies employing the same Bayesian scheme at individual visual field locations. The fact that SORS-Z outperforms SEP supports that SORS can encode and leverage relationships between visual field locations better, without the need of modeling the location relationships explicitly.

### 4.4 Error and estimation bias

**Fig 8:**
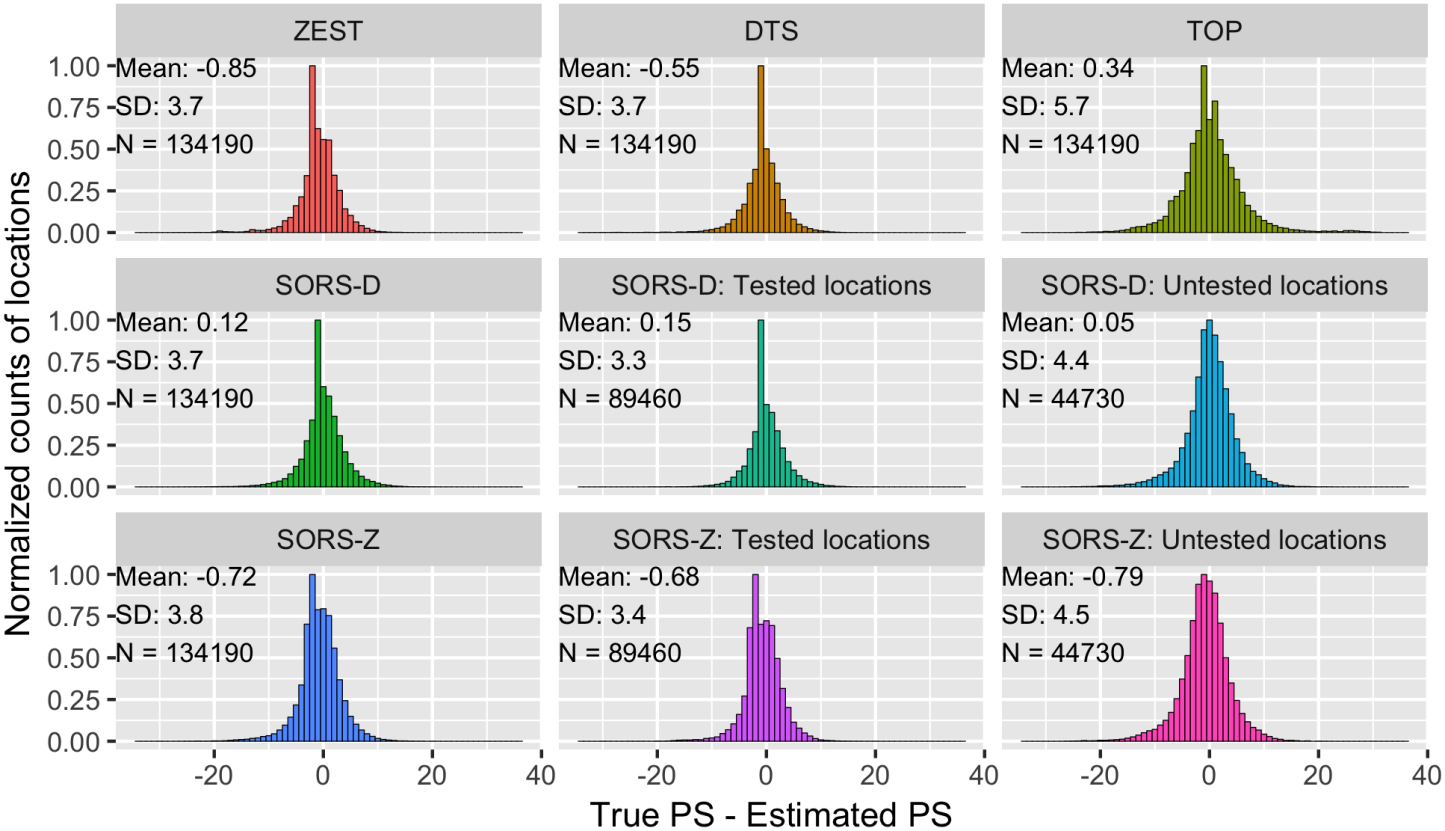
Normalized histogram of signed errors of all visual field locations. Mean, standard deviations (SD) and number of visual field locations (N) per plot are given in the left top corner of each plot. Histograms of errors on tested and untested locations are separately shown for SORS-Z and SORS-D.

To quantify the distribution of errors in the estimation process of the tested perimetry strategies, Fig 8 depicts the histogram of the average signed estimation error per location for ZEST, DTS, SORS-D and SORS-Z. For SORS-Z and SORS-D, we also separately provide error histograms for locations that have been observed and those that have been inferred.

Accordingly, SORS-D leads to the smallest bias when the absolute mean of the distributions is considered. Furthermore, it is biased towards lower values as the mean of the distribution is positive, whereas all other methods except TOP are biased towards higher values. Typically, the tendency to underestimate rather than overestimate PS values is preferable as it is associated with less patient risk. Interestingly, SORS-D uses the same location PS estimation scheme than DTS, yet there is a noticeable reduction in the RMSE. The contribution of SORS is more obvious when DTS is compared to SORS-D at observed locations. This indicates that the way in which SORS selects test locations and estimates the next query stimulus (i.e., the starting estimate of the staircase) is more favorable than that of DTS. As for SORS-Z, it is biased towards higher estimations than the true PS values, showing resemblance to ZEST’s behavior, with a slight reduction in mean and SD.

When we compare the error histograms of untested and tested locations for SORS-D, the bias is reduced with an increase in the standard deviation (SD). This is expected as the variance in the estimation of untested locations is likely to be higher. As expected, SORS-Z has stronger bias towards over-estimation for untested locations than tested locations. The tendency of SORS-Z/ZEST to over-estimate in general is most likely due to sub-optimal configuration of Bayesian PS estimation as discussed in [14]. However, even with sub-optimal parameters, SORS-Z has a comparable and even better performance on average compared to state-of-the-art methods. Moreover, both SORS-Z and SORS-D have preferable error performances compared to TOP which leads to a higher error SD, much higher than SORS’s error SDs at untested locations.

**Fig 9:**
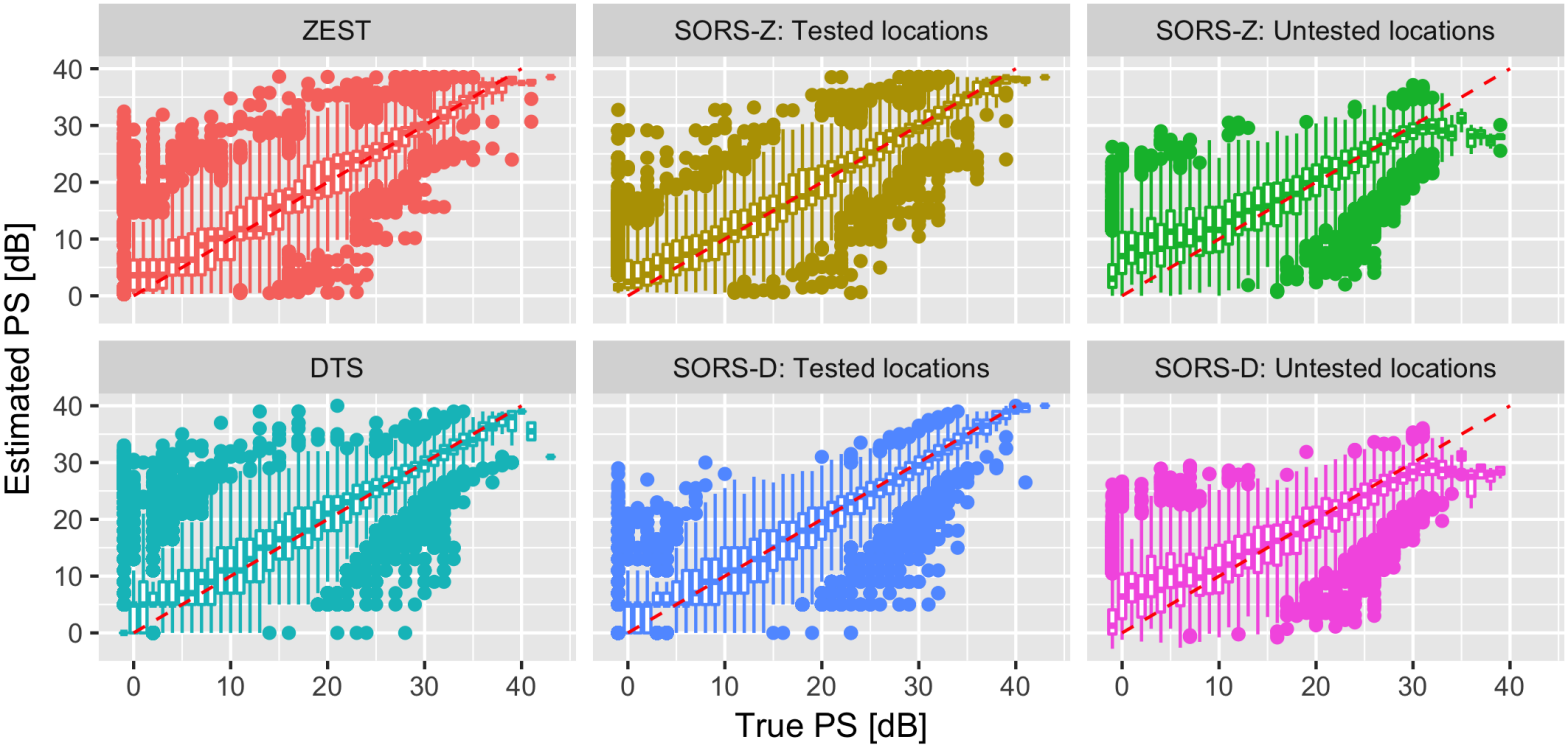
Estimated PS versus true PS for SORS, ZEST and DTS. Estimation bias of SORS techniques in tested and untested locations are shown separately. SORS-D and SORS-Z tested 36 locations.

In Fig 9, we illustrate the estimation bias of the SORS methods with respect to the true PS values found in visual fields, by comparing the predicted PS with the corresponding true values. We again present results of SORS at tested and untested locations. ZEST and SORS-Z have similar estimation bias trends for tested locations. At untested locations, SORS-Z over-/under-estimates at low and high PS values, respectively. SORS-D however suffers from less bias than DTS at tested locations, whereas it also over-estimates in the low-value range of PS when inferring untested locations. In general, the reconstruction procedure that SORS performs for the estimation of non-tested locations results in a smoothed reconstruction, thus avoiding values at both extremes of the dB spectrum.

### 4.5 Performance at scotoma borders

**Fig 10:**
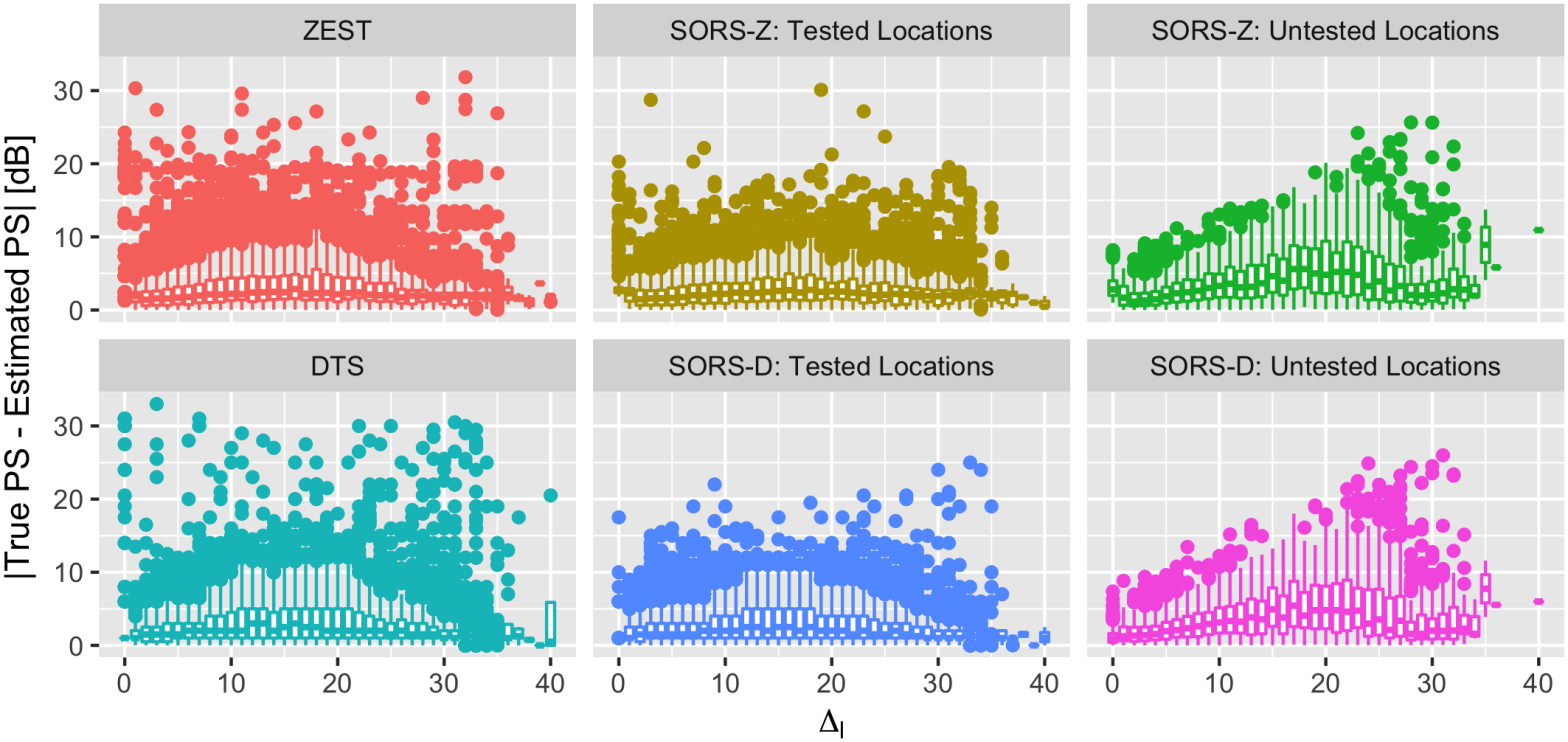
Error performance with respect to Δ_*l*_ per location. Absolute errors are presented for ZEST, DTS and SORS-Z and SORS-D. SORS results are separately shown for tested and untested locations. SORS approaches tested 36 locations.

An important concern with perimetry strategies is their ability to capture scotoma (e.g., regions of isolated impairment). As in [12], we quantify these regions by computing Δ_*l*_ = max_*l*_*n*_ ∈ *N*_*l*__|*t*_*l*_ − *t*_*l*_*n*__| where *t*_*l*_ is the true PS value at a location *l* and *t*_*l*_*n*__ is the true PS of location $l_{n}\in N_{l}$, $N_{l}$ being the set of 8-neighbors of location *l*. Fig 10 depicts the absolute errors, i.e., $|{\hat{t}}_{l}-t_{l}|$ where ${\hat{t}}_{l}$ is the estimated PS value, with respect to Δ_*l*_. Error box plots for tested and untested locations are given separately for SORS-D and SORS-Z. For the error performances on tested locations, SORS-D and SORS-Z show very similar performances with that of ZEST and DTS, while having slightly fewer outliers. For error performances on untested locations, SORS-D and SORS-Z have low median errors in the low and high value range of Δ_*l*_, while they have increased errors in mid-range scotoma values (10 ≤ Δ_*l*_ ≤ 25). Even though, SORS leads to higher median and standard deviations of the errors on untested locations, the majority of errors occur within a reasonable range (i.e., less than 8 dB). Moreover, even for untested locations, both SORS methods lead to less outliers than DTS and ZEST.

### 4.6 Performance dependency on mean deviation

Mean deviation (MD) of a visual field is the average PS deviation from normal reference values collected over a healthy population and is used clinically as an indication of visual impairment. For example, MDs smaller than −2 may signify abnormal eye condition [3].

**Fig 11:**
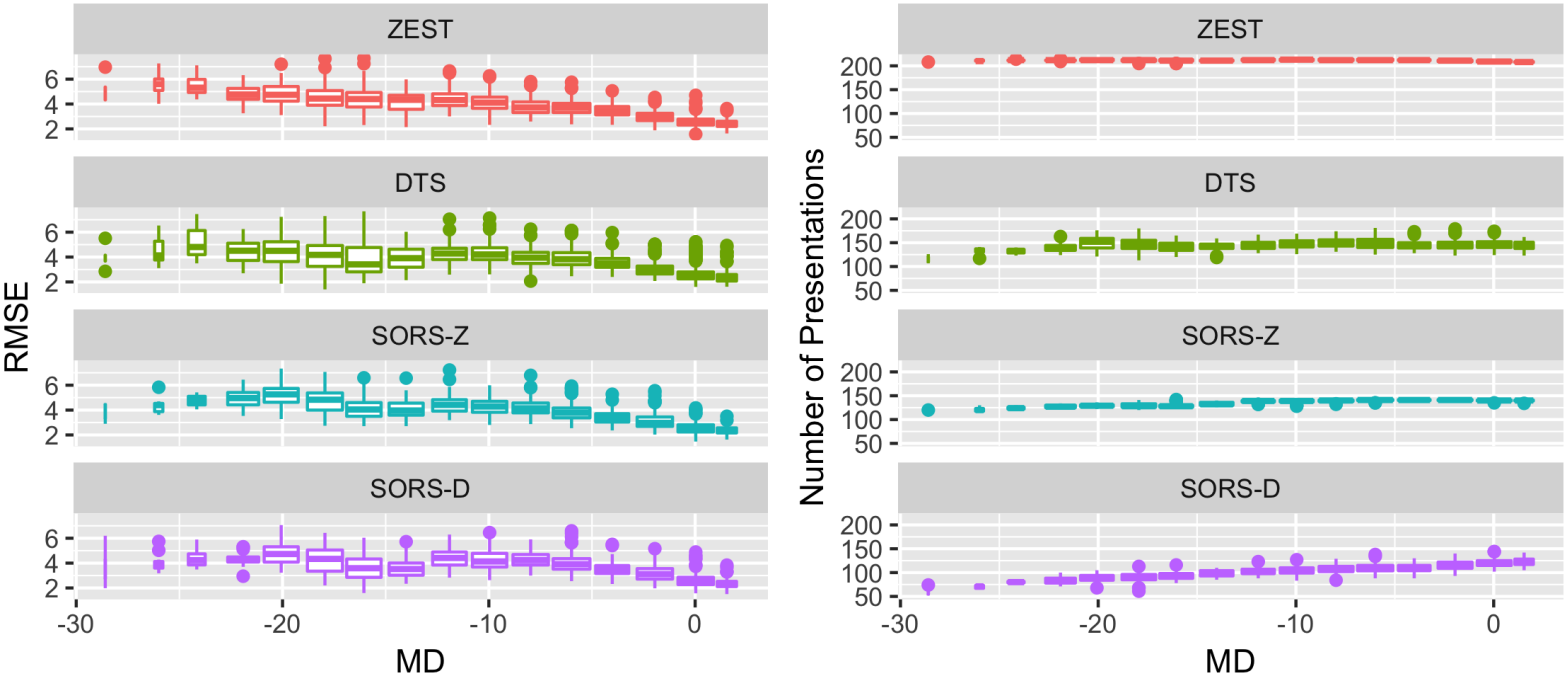
Performance dependency of perimetry strategies on MD in terms of error and speed. We present the dependency of RMSE and number of presentations on MD on the left and right figures respectively. SORS-D and SORS-Z tested 36 locations.

Accordingly, Fig 11 shows the relation between MD and RMSE/speed for all tested strategies. In general, the MD-RMSE relation of each method is similar to one another: small RMSE when MD > −10 and no obvious relation for the rest of the MD range. In terms of number of stimuli presentations, ZEST and DTS have no dependency on MD. Our approaches, especially SORS-D however, appears to depend on MD and surprisingly requires more stimuli for MD > −10. This is due to the fact that within relatively healthy ranges (MD > −10), where SORS-D uses small step sizes in its adaptive staircasing PS estimation method which leads to high precision but slower examinations.

**Fig 12:**
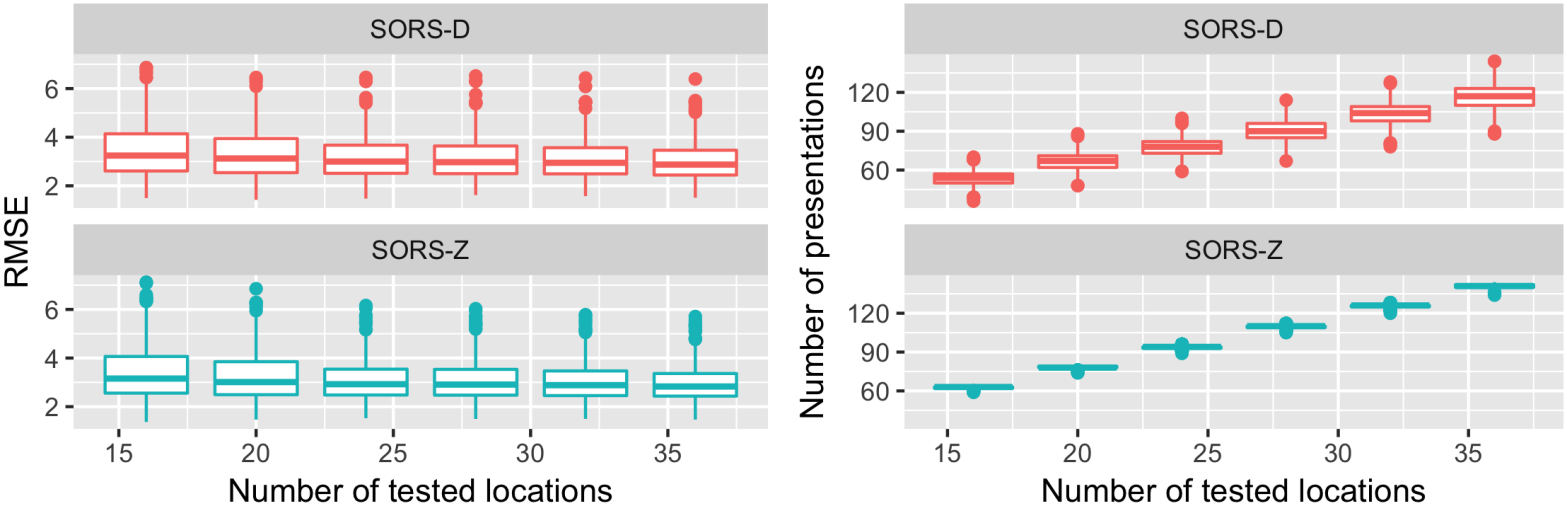
Performance dependency of SORS on the number of tested locations for healthy and early glaucomatous visual fields (MD >−6). We present the dependency of RMSE and number of presentations on MD on the left and right figures, respectively. RMSE slightly changes with the increasing number of tested locations. With approximately 20 locations tested, SORS can double the speed without compromising accuracy.

Fig 12 shows the RMSE and the total number of stimuli presentations with respect to the number of tested locations in SORS-D and SORS-Z for cases of healthy and early glaucomatous visual fields. As can be seen, there is little difference in the average RMSE with respect to number of tested locations. This implies that one can stop SORS earlier for healthier visual fields without compromising accuracy. We also report that the outliers observed over the different number of stimuli presentations are caused by the same visual fields that appear to be harder to estimate.

## 5 Discussion and conclusions

We presented a novel SAP meta-strategy to quickly acquire visual fields as they are currently measured accurately. Our approach leverages the correlations between visual field locations in order to reconstruct the entire visual field from few observed locations. Such a procedure allows our method to be applied at test time in an adaptive way and enables fast convergence to an estimated visual field without having to test all locations. We showed experimentally that SORS speeds up perimetry examination without heavily compromising visual field accuracy and in some cases outperforms state-of-the-art methods outright. This was shown both on healthy and glaucomatous subjects.

While providing better accuracy-speed trade-off, SORS however has some important limitations. SORS is a purely data-driven approach with no parameters to tune except *S*, the number of visual field locations to be tested. As shown in Sec. 4.6, healthier visual fields need fewer number of locations to be tested than glaucomatous visual fields. SORS therefore could be stopped earlier in cases where no further testing is needed. In its current form, SORS does not have an early stopping criterion, therefore it can not adapt to a given visual field at test time. Another limitation of SORS is its deterministic collections of optimal test locations. As shown in Fig 4, the optimized sequence of test locations can differ for healthy or glaucomatous subjects, which could confine its performance. An online procedure for selecting locations during the examination time, e.g., selecting location with high uncertainty as presented in [14] would circumvent such a limitation. In effect, SORS is population-specific in its approach but not patient-specific. These two main limitations are left as open problems that we will explore in the future.

In the future, we plan to investigate how SORS can be made to be tested in batches such that multiple locations are evaluated in parallel as in real examinations. This will allow SORS to be tested on real human subjects, beyond the simulations presented here, which will provide clinical evidence of SORS advantages and limitations. We will also investigate how the importance of different locations can be incorporated into our optimization scheme in order to be more adapted to specific patients or pathologies.

## Appendices

### A Optimization scheme

To illustrate the advantage of our greedy optimization strategy presented in Sec 3, we also compare it to two alternatives in Fig 2. The first is Reconstruction Strategy (RS), where we randomly select *S* in order to build a reconstruction dictionary. The second is Optimized Reconstruction Strategy (ORS), where we select in one step a sequence of *S* locations that minimizes the RMSE among a randomly sampled 50 combinations of *S* locations. Importantly, ORS differs from SORS in that it does not iteratively optimize the location to pick based on the previously selected locations. As seen in Fig 2 (left), SORS-Z outperforms RS-Z and ORS-Z in terms of accuracy-speed trade-off. Similarly, SORS-D outperforms RS-D and ORS-D. One can easily see performance difference between two versions of reconstruction schemes: an algorithm using adaptive staircasing always outperforms its Bayesian PS counterpart. As discussed earlier, this is mainly due to the fact that parameters of Bayesian PS estimation scheme need to be optimized to a specific data set so to perform better than adaptive staircasing.

In the presented RS and ORS in Fig 2 (left), testing scheme is different than SORS: there is no intermediate reconstruction between testing two consecutive locations as in SORS, but reconstruction takes place once after all *S* locations are tested. In this regard, SORS may seem to be advantageous in testing time due to its intermediate reconstruction steps. To remove this testing scheme bias, we incorporated intermediate reconstruction steps into RS and ORS, which we call RSv2 and ORSv2 and compared them to SORS, as presented in Fig 2 (right). Results show that RSv2 and ORSv2 still perform worse than their corresponding SORS versions. This clearly shows that the selection of test locations with associated basis matrices which SORS computes is better optimized than what RS and ORS yield.

### B Test-retest variability

In order to see how much variability our approach induces if the same subject were to be tested multiple times, we tested 5 times the same visual field with SORS-D and SORS-Z. We present distributions of the standard deviations of the PS estimations for both of our approaches as well as for ZEST and DTS in Fig 6. As can be seen from the median SDs, SORS approaches have less test-retest variability than either ZEST or DTS. This result demonstrates the reproducibility of SORS-acquired visual fields, even with certain locations left untested.

### C Performance on sub-populations

Given that not all visual fields are of equal health, Fig 7 (left) and Fig 7 (right) depict the performance results of each method with respect to different populations, namely healthy and glaucomatous patients, respectively. Since glaucomatous samples were abundant in the mixed population set, similar performance was obtained for glaucomatous case as in the mixed population set as was shown in Fig 5 (left). On healthy population however, SORS testing only 16 locations yields to similar accuracy with that of DTS and ZEST with much less number of stimuli presentations.
